# The Effect of *Hedysarum multijugum Maxim*.-*Chuanxiong rhizoma* Compound on Ischemic Stroke: A Research Based on Network and Experimental Pharmacology

**DOI:** 10.1155/2020/6072380

**Published:** 2020-10-06

**Authors:** Kailin Yang, Liuting Zeng, Anqi Ge, Yongmei Shi, Xiaofei Zhu, Wenlong Liu, Jinwen Ge

**Affiliations:** ^1^The First Affiliated Hospital of Hunan University of Chinese Medicine, Changsha, Hunan Province, China; ^2^Hunan University of Chinese Medicine, Changsha, Hunan Province, China; ^3^Capital Medical University, Beijing, China; ^4^Department of Rheumatology and Clinical Immunology, Peking Union Medical College Hospital, Chinese Academy of Medical Sciences & Peking Union Medical College, Beijing, China

## Abstract

**Background:**

*Hedysarum multijugum Maxim.*-*Chuanxiong rhizoma* compound (HCC) is a common herbal formula modified from Buyang Huanwu decoction. Clinical trials have demonstrated its therapeutic potential for ischemic stroke (IS). However, the mechanism of HCC remains unclear.

**Methods:**

The HCC's components were collected from the TCMSP database and TCM@Taiwan database. After that, the HCC's compound targets were predicted by PharmMapper. The IS-related genes were obtained from GeneCards, and OMIM and the protein-protein interaction (PPI) data of HCC's targets and IS genes were obtained from the String database. After that, the DAVID platform was applied for Gene Ontology (GO) enrichment analysis and pathway enrichment analysis and the Cytoscape 3.7.2 was utilized to construct and analyze the networks. Finally, a series of animal experiments were carried out to validate the prediction results of network pharmacology. The expressions of GRP78, p-PERK, and CHOP proteins and mRNAs in different time periods after HCC intervention were detected by Western blot, immunohistochemistry, and RT-qPCR.

**Results:**

A total of 440 potential targets and 388 IS genes were obtained. The results of HCC-IS PPI network analysis showed that HCC may regulate IS-related targets (such as ALB, AKT1, MMP9, IGF1, and CASP3), biological processes (such as endoplasmic reticulum stress, inflammation modules, hypoxia modules, regulation of neuronal apoptosis and proliferation, and angiogenesis), and signaling pathways (such as PI3K-Akt, FoxO, TNF, HIF-1, and Rap1 signaling). The animal experiments showed that HCC can improve the neurobehavioral scores and protect the neurons of IS rats (*P* < 0.05). HCC inhibited the expression of p-PERK in the PERK pathway from 12 h after surgery, significantly promoted the expression of GRP78 protein, and inhibited the expression of CHOP protein after surgery, especially at 24 h after surgery (*P* < 0.05). The results of RT-qPCR showed that HCC can significantly reduce the expression of CHOP mRNA in the neurons in the CA1 region of the hippocampus 72 h after MCAO (*P* < 0.05).

**Conclusion:**

HCC may achieve a role in the treatment of IS by intervening in a series of targets, signaling pathways, and biological processes such as inflammation, oxidative stress, endoplasmic reticulum stress, and angiogenesis.

## 1. Introduction

Globally, stroke is the leading cause of death and disability, and the cost of medical resources after stroke is high [1, 2]. Cerebral infarction (CI) accounts for 70% to 80% of all strokes, while hemorrhagic strokes account for less than 30%. CI, also known as ischemic stroke (IS), is an ischemic necrosis of the brain tissue at the corresponding site after cerebral artery occlusion, which can be complicated by postinfarction hemorrhage [3, 4]. The pathogenesis of IS is thrombosis or embolism, which causes brain tissue ischemia and hypoxia in this blood supply area, leading to nerve cell death and loss of function [4–6]. The symptoms vary depending on the blood vessels involved in the lesion [4]. Current research showed that the main risk factors leading to stroke are diabetes, dyslipidemia, and hypertension, which play an important role in the development of IS [4–6]. The current treatment strategies for IS are mainly (1) acute phase: early improvement of blood circulation in the ischemic area and promotion of neurological recovery; (2) recovery period: continue to strengthen the physical exercise and speech function training of the limbs [7]. In addition to drugs, it can be combined with physical therapy and alternative medicine. Since there is no specific drug, the treatment after IS was still in the focus of research and development. The rise of alternative medicine has gradually provided new strategies for post-IS treatment, especially in developing countries, where cheap and available Chinese herbal medicines are an important choice for patients [8]. Traditional Chinese medicine represented by complex preparation has accumulated a large number of clinical medical treatment practices in the treatment of IS and gradually developed important and effective prescriptions [9]. *Hedysarum multijugum Maxim.*-*Chuanxiong rhizoma* compound (HCC) was first used by the First Affiliated Hospital of Hunan University of Chinese Medicine. It was modified from the classic prescription Buyang Huanwu decoction and has applied for a patented product, which is mainly composed of *Hedysarum multijugum Maxim.*, *Chuanxiong rhizoma*, *Pheretima*, and *Bombyx batryticatus* [10]. Current studies show that HCC can reduce serum TNF-*α* and plasma TXB2 levels and increase plasma 6-keto-PGF1*α* levels in IS patients, suggesting that its therapeutic effect may be related to regulation of vascular endothelial/platelet function balance [11, 12]. In an in vitro model, HCC extract was resistant to rhTNF-*α*-induced apoptosis in cultured HUEC and the effect of rhTNF-*α* on anticoagulant activity. However, its mechanism for treating IS needs further disclosure [13].

**Figure 1:**
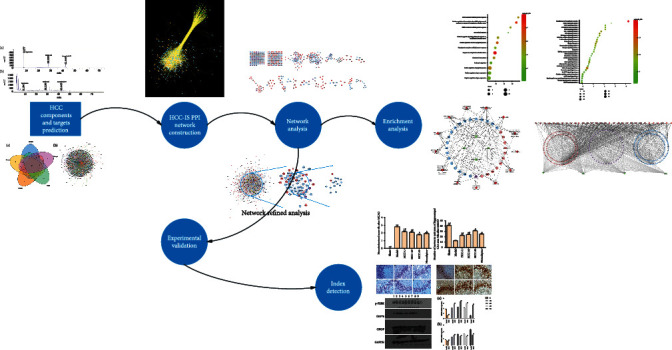
The research processes.

Systematic pharmacological studies have revealed the biological networks in which drugs work. The integration strategy of network biology and multidirectional pharmacology is conducive to expanding the available drug target space and is expected to become one of the new strategies for drug discovery, especially in the field of research and development of Chinese medicine formulae [14–18]. Our previous research successfully analyzed the molecular mechanisms and pharmacodynamic components of traditional Chinese medicine in the fields of cardiovascular and cerebrovascular diseases, tumors, and complex endocrine diseases through the chemical informatics and complex pharmacology methodological strategies [17, 18]. Therefore, in this research, we hope to explore the biological mechanism of endoplasmic reticulum stress after HCC intervention in IS by systematic pharmacology. The research process is shown in Figure 1.

## 2. Materials and Methods

### 2.1. Instruments and Reagents

Instruments being used are as follows: KD2258 paraffin slicer (Zhejiang Jinhua), LEICA DM LB2 binocular microscope (LEICA company, Germany), DNP-9162 electrothermal constant temperature incubator (Shanghai Jinghong Experimental Equipment Co., Ltd.), PCR instrument (2400 PCR system) (PerkinElmer Inc.), Eppendorf benchtop cryogenic microcentrifuge (Eppendorf Inc.), horizontal electrophoresis system (Bio-Rad Inc.), Mixer Gentus (MicroBio), J2-21 high-speed centrifuge, J6-HC centrifuge GS-15R, high-speed benchtop centrifuge (Beckman), ultrapure water meter (American Millipore Company), -80°C ultra-low temperature refrigerator (Zhongke Meiling Cryogenic Technology Co., Ltd.), vertical plate electrophoresis tank DYCZ-24D (Beijing Liuyi Instrument Factory), ultrapure water meter (Millipore company), UV-visible spectrophotometer SP-752 (Spectrum Inc.), desktop high-speed refrigerated centrifuge (Thermo Inc.), medical purification Workbench YJ-875A (Wujiang City Purification Equipment Factory), nucleic acid protein analyzer (Eppendorf Inc.), real-time PCR system (Bio-Rad Inc.), and Motic image advanced 3.2 image analysis system (Motic Company). Column: Welch Ultimate XB-C18 (HS), 4.6 × 250 nm, 5 *μ*m; high-performance liquid chromatograph: Agilent, Germany, Agilent 1260 (diode array detector).

Reagents that were used are as follows: standards: ligustrazine hydrochloride (110817-201608), ferulic acid (110773-201614), astragaloside (110781-201717), all from China Food and Drug Testing Institute. TRIzol was purchased from Invitrogen Inc., USA; RT-PCR kit was purchased from MBI Inc., USA; mRNA Reverse Transcription Kit and SYBR Premix Ex Taq (TAKARA Inc.); HRP-coupled goat anti-rabbit IgG, 2,3,5-triphenylphosphonium tetrachloride (TTC), Tar purple, and SABC kit (SIGMA Inc.); ECL chemiluminescence kit (GE Health Inc.); PV-9000 two-step test kit (Beijing Zhongshan Jinqiao Biotechnology Co., Ltd.); 4% paraformaldehyde (PFA) and phosphate-buffered saline (PBS) (Shanghai Zemai Biotechnology Co., Ltd.); Bradford protein concentration determination kit, hypersensitive chemical emission chromogenic reagent kit, and monoclonal antibody GAPDH (Jiangsu Biyun Tiannonglin Technology Co., Ltd.); SX SDS-PAGE loading buffer, goat anti-mouse secondary antibody, and goat anti-rabbit secondary antibody (Vazyme Biotech Co., Ltd); reverse transcription kit A3500 and real-time PCR mix A6001 (Promega Inc.); DNA marker (Fermentas Inc.); anhydrous ethanol, isopropanol, and chloroform (Shanghai Chemical Reagent Company); primer and gold view nucleic acid dye (Shanghai Shenggong Bioengineering Co., Ltd.); polyclonal antibody GRP78 sc-13968, polyclonal antibody p-PERK (Thr981) sc-32577, and monoclonal antibody GADD153 sc-7351 (B-3) (CHOP) (Santa Cruz Inc.); and diaminobenzidine (DAB) (Beijing Zhongshan Biotechnology Co., Ltd.).

### 2.2. Animal

All experiments were purchased from Hunan Slack Jingda Experimental Animal Co., Ltd. (Changsha, China) and were healthy male specific pathogen-free (SPF) SD rats weighing 200-250 g, animal license number: SYXK (Xiang): 2013-0005. Rats were housed individually in polypropylene cages under conditions of 12-hour light/dark cycle, 26°C room temperature, and 40% relative humidity. Animal experiments were approved by the Animal Ethics Committee of Hunan University of Chinese Medicine (Changsha, China) (Grant No. 201404163) and were in accordance with the National Institutes of Health's *Guide for the Care and Use of Laboratory Animals*.

### 2.3. Preparation of HCC Extract

#### 2.3.1. HCC Extract

HCC is composed of *Hedysarum multijugum Maxim.*, *Chuanxiong rhizoma*, *Pheretima*, and *Bombyx batryticatus* with ratio 4 : 1 : 1.5 : 1.5, which was purchased from the Chinese Pharmacy of the First Affiliated Hospital of Hunan University of Chinese Medicine (Table 1). The herbs were verified by Professor Bing Dai. The HCC extract was prepared by the Department of Pharmacy, the First Affiliated Hospital of Hunan University of Chinese Medicine. Preparation method is as follows: *Chuanxiong rhizoma* 25 g was soaked in 80% ethanol for 45 min and extracted twice (8 times the amount of ethanol; extraction time is 2 h). The extracts were concentrated to a certain consistency under vacuum at 60°C. Hedysarum multijugum Maxim. 100 g, Pheretima 37.5 g, Bombyx batryticatus 37.5 g, and Chuanxiong rhizoma's dregs were soak for 30 min in water and extracted twice (add 10 times of water and extract time, respectively, for 2 h and 1 h); the extracts were concentrated to a certain consistency under vacuum. After that, the extracts were mixed and concentrated to a relative density of 1.30-1.35. Finally, it was dried under vacuum at 60°C to a dry extract of 58.2 g (yield 29.1%) (containing 4 g of crude drug per 1 g of extract powder). The specific experimental procedure refers to our previous study [19]. When in use, the HCC dry extract and physiological saline are formulated into an HCC solution. In this study, the HCC group was divided into the high-, medium-, and low-dose groups, and 15.42 g, 7.71 g, and 3.86 g HCC dry extracts were added to 200 ml of physiological saline, respectively (converted into crude drugs, the concentrations of the high-, medium-, and low-dose groups were 1.8 g/ml, 0.9 g/ml, and 0.45 g/ml, respectively). Nimodipine tablets were purchased from Zhengzhou Ruikang Pharmaceutical Co., Ltd. (20 mg/tablet), dissolved in physiological saline at 3.2 mg/ml, and then filtered and stored at 4°C.

#### 2.3.2. High-Performance Liquid Chromatography

To verify the quality of HCC, we qualitatively verified three representative compounds in HCC using high-performance liquid chromatography (HPLC): astragaloside IV, ferulic acid, and ligustrazine (conditions: ultimate XB-C18 column (5 *μ*m, 4.6 × 250 mm); A: acetonitrile; B: 0.2% phosphoric acid-water; gradient elution flow rate: 1 ml/min; detection wavelengths: 198 nm, 201 nm, 280 nm, 290 nm, 315 nm, and 320 nm; injection volume: 10 *μ*l) (Table 2).

**Figure 2:**
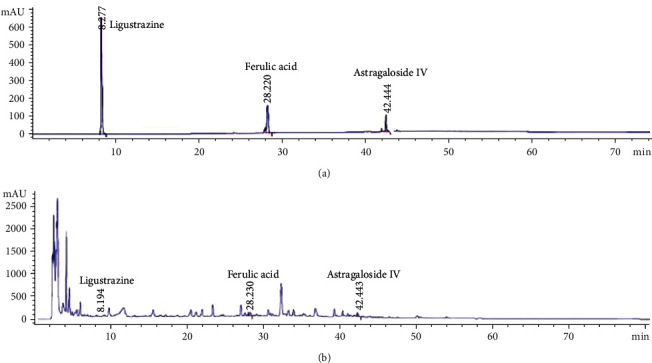
HCC's fingerprint ((a) mixed standard and (b) HCC).

The HPLC of HCC is shown in Figure 2. The main compounds of HCC were quantified: astragaloside IV 13.72 mg/200 g, ferulic acid 2.52 mg/200 g, and ligustrazine 0.36 mg/200 g.

### 2.4. Prediction of the Mechanism of HCC Intervention in Ischemic Stroke by Network Pharmacology

#### 2.4.1. HCC's Potential Compound Prediction

The Traditional Chinese Medicine Systems Pharmacology Database (TCMSP) (http://tcmspw.com/tcmsp.php) [20], Traditional Chinese Medicine Database@Taiwan (TCM@Taiwan) (http://tcm.cmu.edu.tw/zh-tw/) [21], and Traditional Chinese Medicines Integrated Database (TCMID) (http://119.3.41.228:8000/tcmid/) [22] were utilized to collect all compounds of *Hedysarum multijugum Maxim.*, *Chuanxiong rhizoma*, *Pheretima*, and *Bombyx batryticatus*. Oral bioavailability (OB), drug-likeness (DL), and Caco-2 permeability were utilized to identify the potential bioactive compounds of HCC [17, 18, 23–26]. The compounds that meet the standards of OB ≥ 30%, Caco‐2 > −0.4, and DL ≥ 0.18 were regarded as oral absorbable compounds with biologically active [17, 18, 23–26].

Since the use of the ADME model to predict the potential compounds of HCC has limitations [27], in order to avoid the omission of potential compounds, we searched a large number of references and included oral absorbable compounds with pharmacological activity. Combined with relevant references [28, 29], several compounds are included.

#### 2.4.2. Potential Targets of HCC

The structure of the potential compounds of HCC is drawn in ChemBioDraw Ultra 12.0 software and stored in mol2 format. Their molecular structures were confirmed using SciFinder (http://scifinder.cas.org), PubChem (https://pubchem.ncbi.nlm.nih.gov/), TCMSP, ChemSpider (http://www.chemspider.com), and Chemical Book (http://www.chemicalbook.com/).

The mol2 format file of the potential compounds molecular structure was imported into PharmMapper (http://lilab-ecust.cn/pharmmapper/) to predict the potential target of HCC [30]. The PDB ID of the HCC protein target is introduced into the UniProt database (https://www.uniprot.org/uniprot/) with the species limited to “Homo sapiens” to search for the official symbols of each protein target (Table S1, see Supplementary Materials).

#### 2.4.3. Ischemic Stroke Gene

To construct the biological network of IS, the IS-related genes were collected from the OMIM database (http://omim.org/) [31] and GeneCards (http://www.genecards.org) [32]. Finally, one thousand and six hundred and thirty-five (1635) IS-related genes were obtained. These IS-related genes with relevance score > 2.5 were used for subsequent biological network construction and network analysis (Table S2).

#### 2.4.4. Protein-Protein Interaction Data

The String database (https://string-db.org/, Version 11) is a database containing known and predicted protein-protein interaction (PPI) data [33]. The potential targets of HCC were introduced into the String database with the species limited to “Homo sapiens” and the PPI data of them were obtained. The result is saved in TSV format. The node 1, node 2, and combined score information in the file is retained and imported into Cytoscape software to draw the interaction network [17, 18, 34].

#### 2.4.5. Network Construction and Analysis

The Cytoscape software ver. 3.7.0 was used for network construction. In Cytoscape, each node represents a gene, protein, or molecule, and the node-to-node relationship (edges) represents the interaction between these biomolecules. The degree of the node represents the number of nodes connected to the node in the network. The greater the degree is, the more likely this target becomes the key target in the network [34].

In the interaction network, there are several regions where proteins interact closely; such regions are called cluster [34]. These different clusters represent different biological modules, and it is possible to treat diseases by intervening in these modules. The plugin of Cytoscape, MCODE, was utilized to detect clusters in PPI network [17, 18].

DAVID (https://david.ncifcrf.gov/summary.jsp, Version 6.8) provides systematic, comprehensive biofunctional annotation information for large-scale genes or proteins to identify the most significant enriched biological annotations. This database was utilized to undergo Gene Ontology (GO) enrichment analysis and pathway enrichment analysis for these networks [35].

### 2.5. Experimental Methods

#### 2.5.1. Model Preparation

Modeling was carried according to the improved Zea Longa intraluminal tether block method [36]. The production of the wire plug is as follows: the diameter of the monofilament nylon wire is 0.22-0.24 mm, and each segment is cut into a length of 4-5 cm; disinfect the line with alcohol and soak it in heparin sodium. The rat focal cerebral ischemia model was prepared by the middle cerebral artery occlusion (MCAO) method: (1) anesthesia: adult SD rats were anesthetized by intraperitoneal injection of 4% sodium pentobarbital (50 mg/kg); (2) fixation and disinfection: after complete anesthesia, the rat is placed in a supine position on a rat fixation plate, sheared, and the skin is disinfected with complex iodine; (3) incision: take the midline incision of the neck, generally 2.5-3.5 cm; (4) isolation and ligation of blood vessels: the right sternocleidomastoid muscle was bluntly separated, and the right common carotid artery (CCA) was separated with forceps and the proximal end of CCA was ligated; locate the CCA bifurcation and separate the inwardly communicating external carotid artery (ECA) and the outwardly moving internal carotid artery (ICA), ligature the branch of ECA, and then ligature the ECA; (5) place the line: the ICA was clamped with an arterial clip, and then a small opening was made 4 mm below the CCA bifurcation. The artery clamp was released and the iliac line (0.28 mm diameter fishing line, head end 0.34 mm) was inserted into the ICA with an insertion depth of 22 ± 0.5 mm. Finally, the telecentric end of ICA and CCA is ligated; and (6) stitching: cut off the extra tying line, sprinkle a small amount of penicillin powder in the incision, suture the incision layer by layer, and clean the incision. The blank group does not insert the thread plug, and the remaining steps are the same as the model group. After anesthesia was awoken, the rat's eyelids drooped, showing a significant Horner syndrome. Then TTC staining was used to judge whether the modeling was successful. After the operation, the rats were kept in a single cage, and the breeding room was kept quiet and constant temperature and humidity.

#### 2.5.2. Animal Grouping and Intervention Methods

Two hundred and seventy (270) healthy male SPF SD rats were randomly divided into 6 groups (45 in each group): the blank group (the sham operation group), the model group (the IS group), the HCC low-dose group (4.5 g crude drug/kg), the HCC medium-dose group (9 g crude drug/kg), the HCC high-dose group (18 g crude drug/kg), and the nimodipine group (32 mg/kg). The MCAO model was prepared for each of the other groups except the blank group. Each group was given the corresponding drug by oral gavage per 100 g body weight once a day 7 days before surgery, and the model group was given normal saline (NS). The drug was administered continuously 7 days before the modeling, and the last time was 1 hour before the modeling. The HCC low-, medium-, and high-dose groups were given 4.5 g crude drug/kg, 9 g crude drug/kg, and 18 g crude drug/kg concentration of HCC extract, respectively.

#### 2.5.3. Neurobehavioral Score

Neurobehavioral scores are based on the Zea Longa 4-point scale [36]. Neurobehavioral examination was divided into 5 grades: 0 point: normal, no neurological deficit; 1 point: the left front paw cannot be fully extended, mild neurological deficit; 2 points: when walking, the rat turned to the left side (temporal side), moderate neurological deficit; 3 points: when walking, the rats were dumped to the left side (temporal side), and severe neurological deficits were observed; and 4 points: cannot walk, lose consciousness.

#### 2.5.4. Morphological Observation

Nissl staining was performed 72 hours after MCAO as follows: rats were anesthetized with 4% pentobarbital sodium at a dose of 50 mg/kg, the abdominal wall was cut open, the diaphragm was exposed, and the diaphragm and chest wall and pericardium were carefully cut open to reveal the heart. Use a perfusion needle to puncture the left ventricle through the apex to the root of the aorta, then fix the puncture needle with a hemostatic forceps and cut a small opening in the right atrial appendage until the blood is seen to flow out. Then, use a tee to inject 37°C NS (infused with 0.5 l/kg body weight); after the liquid flowing out of the right atrial appendage became clear, it was perfused with 4% paraformaldehyde of 4°C in 0.1 mol/l phosphate buffer for about 25-30 minutes. The rat hemispheres were placed in 4% paraformaldehyde in a refrigerator at 4°C for 24 h, embedded in OCT gel, coronally sectioned with a cryostat (the thickness was 20 *μ*m), placed in antifreeze buffer, and attached to gelatin slides with a brush in order. The patch was immersed in 70% alcohol for 4 h and washed for 3 min, dyeing with 0.1% CV for 10-15 min (determination of staining time under microscope), washing with double distilled water, and drying. Then, dehydrated with 100% alcohol, fixed twice with xylene (10 min each time), and sealed with neutral gum. Light microscopy was used to observe the histomorphological changes in the ischemic area (especially the hippocampus) (including whether the cells were atrophied or not and whether the Nissl was dissolved).

#### 2.5.5. Immunohistochemical Detection of Rat Hippocampal Tissue Regulatory Protein (GRP) 78 and c/EBP Family Homologous Protein (CHOP) Protein Expression

Four rats in each group were anesthetized at 6 h, 12 h, 24 h, 48 h, and 72 h after MCAO, opening the chest, and perfusion, and the brain tissue was fixed in 4% paraformaldehyde at 4°C for 24 h. The tissue was then sequentially invaded into 10%-20%-30% sucrose solution (the thickness was 20 *μ*m) and sliced continuously in a horizontal section and placed in 0.1 M PBS buffer.

Immunohistochemical procedure is as follows: the specimens were treated with 3% H_2_O_2_ (methanol) for 15 minutes, washed with 0.01 M PBS for 3 times (5 min each), and blocked with 5% BSA (normal fetal bovine serum) for 1 h, and drain the BSA. Then, 1 : 200 rabbit anti-GRP78 polyclonal antibody, 1 : 100 rabbit anti-p-PERK polyclonal antibody, and 1 : 200 mouse anti-GADD 153 (CHOP) monoclonal antibody were added, incubated at 4°C for 24 h, and washed with 0.01 M PBS for 3 times (10 min each). Add the corresponding biotinylated immunoglobulin IgG (goat anti-rabbit secondary antibody and rabbit anti-mouse secondary antibody 1 : 1000), incubate for 1.5 h at room temperature, and wash with 0.01 M PBS for 3 times (10 min each). After that, add streptavidin-biotin-horseradish peroxidase complex (SABC), incubate for 1.5 h at room temperature, and wash with 0.01 M PBS for 3 times (10 min each). Then, DAB was used to develop color (0.05%DAB + 0.01%H_2_O_2_ in the dark) for 3-5 min, and the reaction time was controlled by microscopic observation. The reaction was terminated by adding 0.01 M PBS at 4°C, naturally dried, dehydrated with a gradient alcohol, transparentized with xylene, and sealed with a neutral gum. The negative control group used 0.01 M PBS instead of the primary antibody to exclude nonspecific staining of the secondary antibody.

Gray value analysis is as follows: six slices were randomly taken from each animal, and six fields were randomly taken under a 20-fold objective lens. The image was processed by the Motic image analysis system, and the gray value of each field of view was measured and calculated. The average gray value of each group of animals was compared (gray value range 0-255; the larger the gray value is, the lower the expression level of the positive product is).

#### 2.5.6. Western Blot Detection

The rat hippocampus was taken, homogenized by adding 5 volumes of buffer, and centrifuged, and the supernatant was taken. The protein concentration was quantified using the Lowry method protein and then subjected to sodium dodecyl sulfate-polyacrylamide gel electrophoresis (SDS-PAGE). The order of the sampling was as follows: the IS group 6 h after surgery (M6), 12 h after surgery (M12), 24 h after surgery (M24), and 48 h after surgery (M48); the HCC group 6 h after surgery (HCC6), 12 h after surgery (HCC12), 24 h after surgery (HCC24), and 48 h after surgery (HCC48).

Immunoassay is described as follows: the target nitrocellulose membrane was placed in a plastic bag of moderate size, and a blocking solution (3% BSA+5% skim milk) was added and shaken slowly for 1 h at room temperature, and was washed 3 times with 0.02 M PBS for 10 min each time. Then, 1 : 500 rabbit anti-GRP78 polyclonal antibody, rabbit anti-p-PERK polyclonal antibody, mouse anti-GADD 153 (CHOP) monoclonal antibody, and 1 : 1000 mouse anti-GAPDH monoclonal antibody 5-8 ml were added in an amount of 0.1 ml/cm^2^ according to the membrane area and incubated at 4°C overnight. The membrane was taken out the next day and washed 3 times with 0.01 M PBS for 10 min each time; continue to add horseradish peroxidase-labeled goat anti-rabbit or goat anti-mouse secondary antibody (1 : 1000) according to the membrane area in an amount of 0.1 ml/cm^2^ and incubate for 1.5 h at room temperature; after that, the membranes were washed 3 times with 0.02 M PBS for 10 min each time. Subsequently, color development, exposure, and photography were carried out using an electrochemical-enhanced luminescence (ECL) immunoassay kit. The results were analyzed by IPP6.0 image analysis system, and the corresponding IOD value and the ratio of glyceraldehyde-3-phosphate dehydrogenase (GADPH) were obtained. Protein expression was positively correlated with IOD values.

#### 2.5.7. RT-qPCR Detection of Related Gene Expression

The total mRNA was extracted from the tissues extracted with TRIzol, and the RNA purity was identified by 1.2% agarose gel electrophoresis. Subsequently, the first-strand cDNA synthesis was carried out: cDNA was synthesized by reverse transcription using the total tissue mRNA as a template, and the obtained cDNA was stored at -20°C. Then, a real-time PCR test is performed. The amplification procedure is described as follows: 94°C for 4 min, 94°C for 40 sec, and 60°C for 30 sec; the CT value was obtained, and the relative expression amount of the target gene was calculated by the 2^-*ΔΔ*CT^ method. QT-PCR primer sequence is described as follows: the gene sequence was downloaded from the NCBI database. The qPCR primers were designed using Primer 5.0 software. The primer sequences are shown in Table 3.

### 2.6. Statistical Analysis

All data are expressed as the mean ± SD. Statistical analysis was performed using SPSS 19.0 software. For multiple comparisons, one-way analysis of variance (ANOVA) was performed and the comparison between the two groups was analyzed using the LSD test. *P* < 0.05 was considered to be statistically significant.

## 3. Results and Discussion

### 3.1. HCC's Potential Compounds and Targets and IS Genes

According to the standard (OB ≥ 30%, Caco‐2 > −0.4, and DL ≥ 0.18), a total of 32 compounds were included: sitosterol, wallichilide, 7-O-methylisomucronulatol, isoflavanone, 1,7-dihydroxy-3,9-dimethoxy pterocarpene, 3,9-di-O-methylnissolin, ergotamine, hederagenin, isorhamnetin, xanthinin, mairin, perlolyrine, bassianin, (6aR,11aR)-9,10-dimethoxy-6a,11a-dihydro-6H-benzofurano[3,2-c]chromen-3-ol (73340-41-7), bifendate, lupeol acetate, calycosin, kaempferol, senkyunone, (3S,8S,9S,10R,13R,14S,17R)-10,13-dimethyl-17-[(2R,5S)-5-propan-2-yloctan-2-yl]-2,3,4,7,8,9,11,12,14,15,16,17-dodecahydro-1H-cyclopenta[a]phenanthren-3-ol (64997-52-0), hyrcanoside, mandenol, 4-guanidino-1-butanol, cholesteryl ferulate, (3R)-3-(2-hydroxy-3,4-dimethoxyphenyl)chroman-7-ol (64474-51-7), ecdysterone, quercetin, guanosine, jaranol, beauvericin, myricanone, and formononetin. By searching the literature and combined with HPLC results, a total of 10 oral absorbable compounds with pharmacological activity were added: ligustrazine, senkyunolide H, ononin, calycosin 7-O-glucoside, butylphthalide, senkyunolide I, senkyunolide A, coniferyl ferulate, chlorogenic acid, and astragaloside IV.

**Figure 3:**
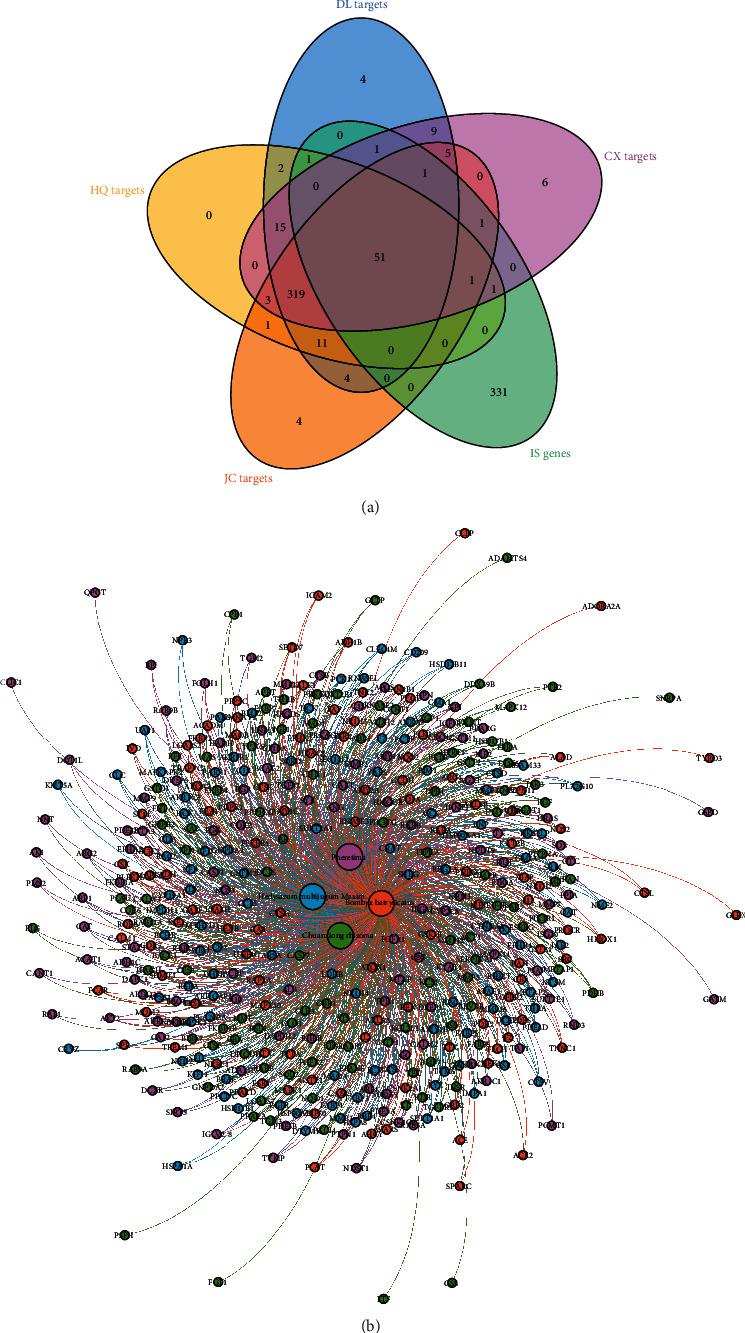
(a) Venn diagram of potential targets and IS genes. (b) Herb-potential target network (HQ: *Hedysarum multijugum Maxim.*; CX: *Chuanxiong rhizoma*; DL: *Pheretima*; JC: *Bombyx batryticatus*).

After the potential target prediction, totally, 440 potential targets were obtained. *Hedysarum multijugum Maxim.* contains 405 potential targets, *Chuanxiong rhizoma* contains 413 potential targets, *Pheretima* contains 423 potential targets, and *Bombyx batryticatus* contains 401 potential targets. Meanwhile, after searching, a total of 1635 IS-related genes were obtained. These IS-related genes with relevance score > 2.5 were used for subsequent biological network construction and network analysis. There is overlap between the target sets (Figure 3(a)). The relationship among herbs and targets is shown in Figure 3(b).

### 3.2. HCC-IS PPI Network Analysis

#### 3.2.1. HCC-IS PPI Network

**Figure 4:**
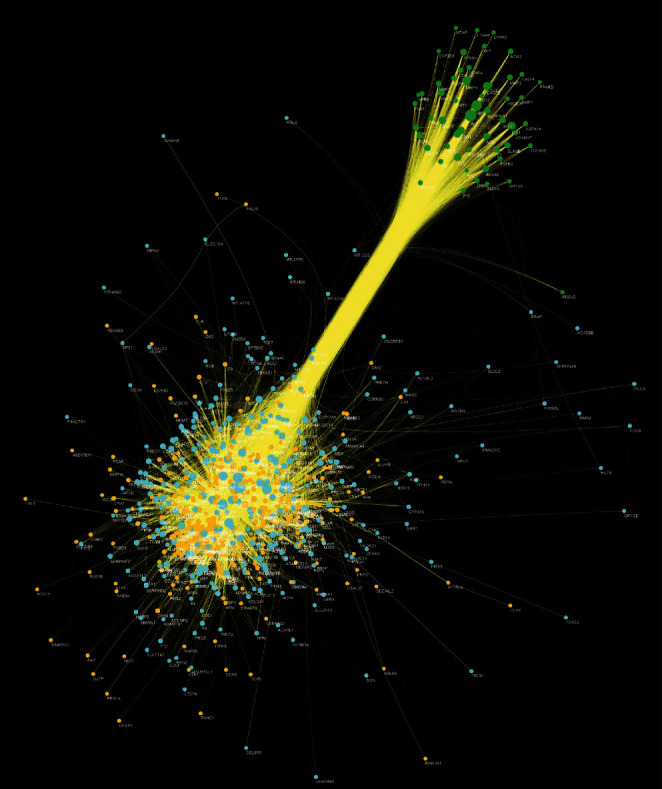
HCC-IS PPI network (green circle stands for HCC-IS; blue circle stands for IS genes; orange circle stands for HCC targets).

The relationship among HCC's potential targets and IS genes was shown in HCC-IS PPI network. This network is composed of 56 HCC-IS targets, 373 HCC targets, 299 IS targets, and 17142 edges. The top 20 targets of high degree are selected and divided into three categories: (1) IS genes: INS (328 edges), IL6 (305 edges), TNF (276 edges), VEGFA (270 edges), TP53 (244 edges), EGF (230 edges), CXCL8 (208 edges), IL10 (189 edges), CCL2 (183 edges), IL1B (182 edges), and TLR4 (176 edges); (2) HCC targets: EGFR (217 edges), MAPK1 (207 edges), SRC (204 edges), and MAPK8 (182 edges); and (3) HCC-IS targets: ALB (341 edges), AKT1 (295 edges), MMP9 (214 edges), IGF1 (194 edges), and CASP3 (188 edges) (Figure 4).

#### 3.2.2. Biological Processes of HCC-IS PPI Network

**Figure 5:**
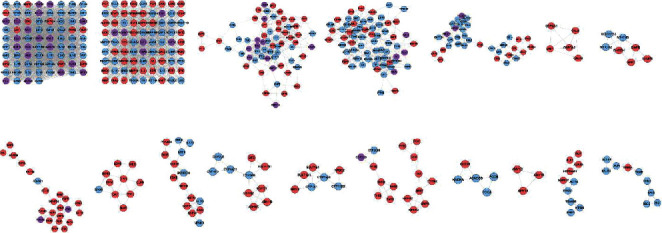
Cluster of HCC-IS PPI network (purple circle stands for HCC-IS; blue circle stands for IS genes; pink circle stands for HCC targets).

The HCC-IS PPI network was analyzed by MCODE to obtain the clusters. The clusters of this network are shown in Figure 5 and Table 4. The genes and targets in these clusters were put into the DAVID database to undergo GO enrichment analysis as an example.

After the GO enrichment analysis, a lot of biological processes of each cluster were returned. Cluster 1 is associated with inflammation modules, hypoxia modules, regulation of neuronal apoptosis and proliferation, angiogenesis, coagulation and platelet activation, oxidative stress, and so on. Cluster 2 is associated with coagulation, inflammation, hypoxia, angiogenesis, endothelial cell proliferation, response to estrogen, and nitric oxide biosynthesis. Cluster 3 is involved in the regulation of coagulation, fibrinolysis, negative regulation of endothelial cell apoptosis, oxidative stress, neuronal production, and regulation of vascular and inflammatory responses. Cluster 4 is related to endoplasmic reticulum stress and neuronal apoptosis. Cluster 5 is related to lipid synthesis and metabolism such as cholesterol and triglycerides. Cluster 8 is involved in the metabolism of lipids. Cluster 10 is related to redox reactions such as glutathione. Cluster 12 is associated with the metabolism of steroid hormones. Cluster 13 is associated with the metabolism of steroid hormones. Cluster 15 is related to lipid metabolism. Cluster 18 is related to endoplasmic reticulum stress. Clusters 6, 9, 11, 14, and 16 did not return any IS-related biological processes. Cluster 7 failed to return any human biological processes (Table S3).

**Figure 6:**
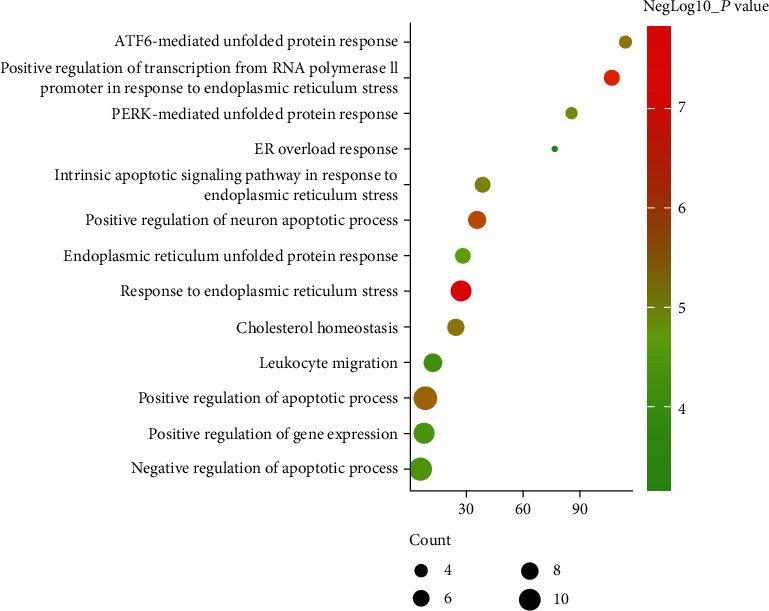
Bubble chart of biological processes of Cluster 4 (*x*-axis stands for fold enrichment).

**Figure 7:**
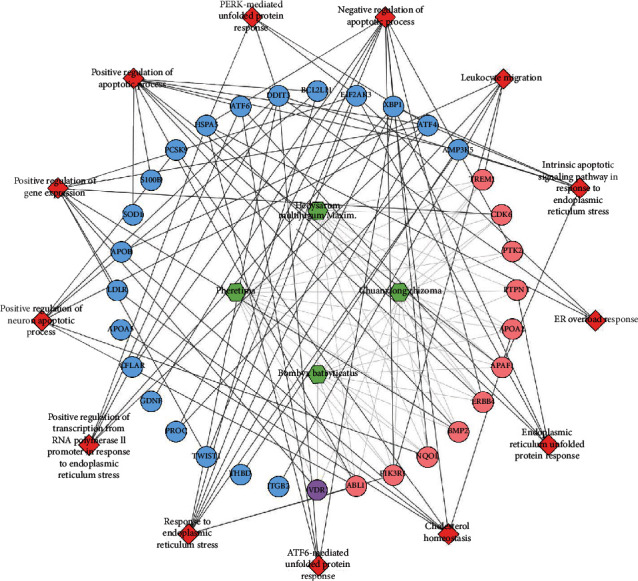
Herb-biological processes-target network (red diamond stands for biological processes; purple circle stands for HCC-IS; blue circle stands for IS genes; pink circle stands for HCC targets. The gray lines stand for the relationship among herbs and targets; the black lines stand for the relationships among biological processes and targets).

Since Cluster 4 contains many classic biological processes, bubble chart is created using the main biological process data contained in Cluster 4 (Figures 6 and 7).

In summary, through the analysis of the HCC-IS PPI network, we reveal the potential mechanism of HCC treatment of IS, which is mainly related to inflammation, hypoxia, endoplasmic reticulum stress, oxidative stress, angiogenesis, coagulation and platelet activation, angiogenesis, endothelial damage, the negative regulation of endothelial cell apoptosis, and the regulation of angiogenesis and inflammatory response after IS. These biological processes occur mainly in the early stages of IS [37–40]. These directions can be developed in the future when researching IS treatment strategies.

#### 3.2.3. Signaling Pathways of HCC-IS PPI Network

**Figure 8:**
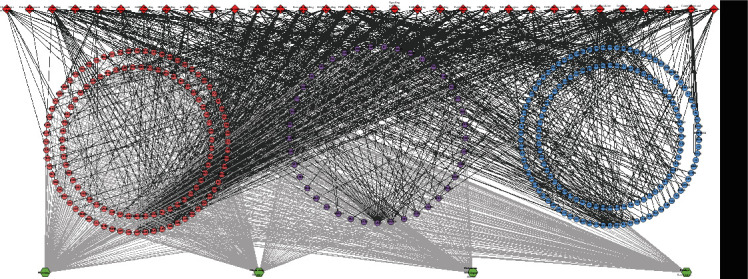
Signaling pathway of HCC-IS PPI network (red diamond stands for signaling pathway; purple circle stands for HCC-IS; blue circle stands for IS genes; pink circle stands for HCC targets. The gray lines stand for the relationship among herbs and targets; the black lines stand for the relationships among pathways and targets).

The HCC targets combining with IS genes were put into the DAVID database for pathway enrichment analysis. After this, thirty-two (32) IS-related signaling pathways were returned (Figure 8).

**Figure 9:**
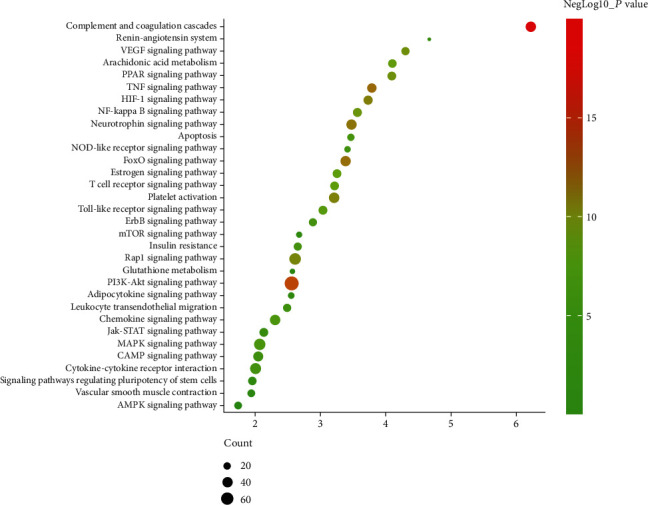
Bubble chart of the signaling pathway (*x*-axis stands for fold enrichment).

These signaling pathways are ranked according to the degree of enrichment (negative correlation with *P* value) and count from large to small. The top 10 is the complement and coagulation cascades, PI3K-Akt signaling pathway, TNF signaling pathway, FoxO signaling pathway, neurotrophin signaling pathway, HIF-1 signaling pathway, platelet activation, Rap1 signaling pathway, and VEGF signaling pathway (Figure 9 and Table S4).

The signaling pathway enrichment shows that these signaling pathways are closely related to the pathological changes of IS. For example, it is involved in the signaling pathways such as complement and coagulation cascades, and platelet activation that occur in the early stage of IS [41]. The PI3K-Akt signaling pathway is associated with inflammation, endoplasmic reticulum stress, and neuronal apoptosis in neurovascular units [42]. The FoxO signaling pathway is related to endoplasmic reticulum stress and oxidative stress [43]. The TNF signaling pathway and NF-*κ*B signaling pathway are related to inflammation [44]. The HIF-1 signaling pathway is related to angiogenesis [45]. The neurotrophin signaling pathway is associated with neural stem cell differentiation and synaptic regeneration [46]. The PPAR signaling pathway is related to energy metabolism [47]. After IS, neurovascular units (which includes the neurons, blood-brain barrier, microglia, and extracellular matrix components; there is a wide range of signal connections between them, which is the material basis for ensuring neuron function and normal cerebral blood flow) play a very important role in the pathophysiology and clinical treatment of IS [48–50]. The neurons, blood-brain barrier, and microglia are intertwined through various cascade response signaling pathways after ischemia and hypoxia to jointly promote the occurrence, development, and outcome of IS [51, 52].

**Figure 10:**
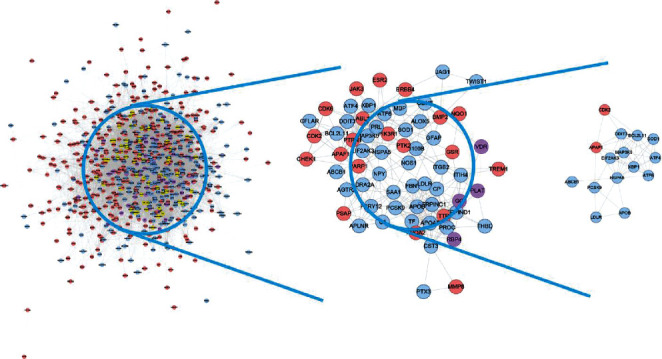
The main core targets related to the endoplasmic reticulum stress.

In summary, this study explored the mechanism of HCC intervention in IS through network pharmacology strategies; in order to further reveal the specific biological processes and targets related to the therapeutic effect of HCC, we conducted MCODE analysis on the HCC-IS PPI network to obtain clusters of biological modules. After network analysis and literature mining, we found that there are few studies on the role of endoplasmic reticulum stress biological modules in IS (Figure 10). In this study, endoplasmic reticulum stress is one of the most important biological modules in the HCC-IS PPI network, and its cluster ranks fourth. The biological modules related to the first three clusters (inflammation, angiogenesis, etc.) have been studied a lot, and there are related therapeutic drugs. Therefore, this study will use IS animal model experiments to verify and study the main core targets [GRP78 (HSPA5), p-PERK (EIF2AK3), and CHOP (DDIT3)] in the endoplasmic reticulum stress biological module.

### 3.3. Neurobehavioral Score

**Figure 11:**
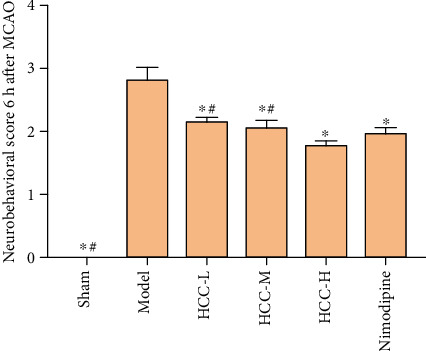
Neurobehavioral score (6 h after MCAO. ^∗^Compared with the model group, *P* < 0.05. ^#^Compared with the HCC high-dose group, *P* < 0.05).

The results showed that the neurobehavioral scores of the model group were significantly higher than those of the sham operation group (*P* < 0.05). Compared with the model group, each dose of HCC and nimodipine can significantly reduce the neurobehavioral score of rats after MCAO (*P* < 0.05). Compared with the low-dose group and the middle-dose group, the HCC high-dose group was more effective, and the difference was statistically significant (*P* < 0.05) (Figure 11).

### 3.4. Morphological Observation

**Figure 12:**
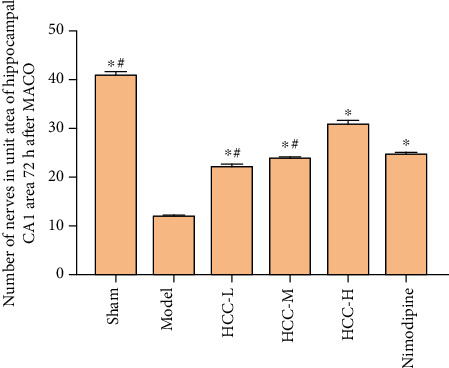
Number of the neurons in unit area of hippocampal CA1 area (72 h after MCAO. ^∗^Compared with the model group, *P* < 0.05. ^#^Compared with the HCC high-dose group, *P* < 0.05).

**Figure 13:**
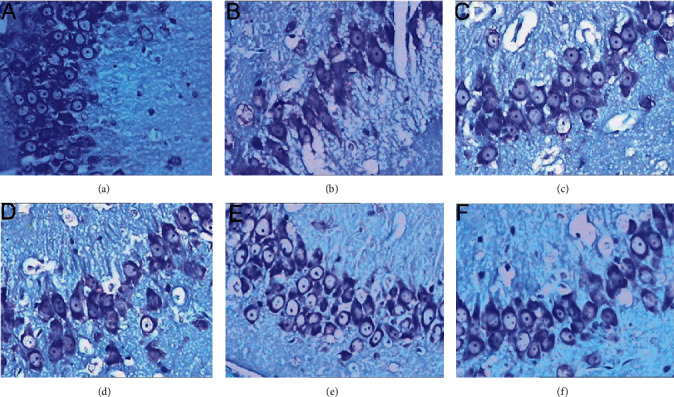
Nissl staining in the hippocampal CA1 area (X400) (72 h after MCAO. (a) The sham operation group. (b) The model group. (c) The HCC low-dose group. (d) The HCC medium-dose group. (e) The HCC high-dose group. (f) The nimodipine group).

Nissl staining showed that the Nissl body was located in the cytoplasm and was purple-red. Compared with the model group, the HCC high-, medium-, and low-dose groups and the nimodipine group had clear cell boundaries, and the cytoplasmic Nissl bodies were evenly distributed, and the nucleoli were clearly visible. HCC and nimodipine can significantly increase the number of the neurons surviving per unit area of the hippocampal CA1 area, and the difference is statistically significant; and compared with the low-dose group and the middle-dose group, the HCC high-dose group was more effective (*P* < 0.05) (Figures 12 and 13).

### 3.5. The Expressions of GRP78, p-PERK, and CHOP Protein Detected by Immunohistochemistry

#### 3.5.1. Expression of GRP78 Protein

**Figure 14:**
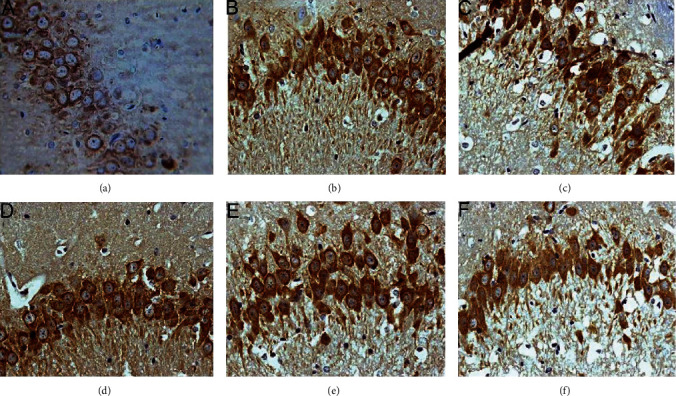
Expression of GRP78 protein in each group (24 hours after intervention. (a) The sham operation group. (b) The model group. (c) The HCC low-dose group. (d) The HCC medium-dose group. (e) The HCC high-dose group. (f) The nimodipine group).

**Figure 15:**
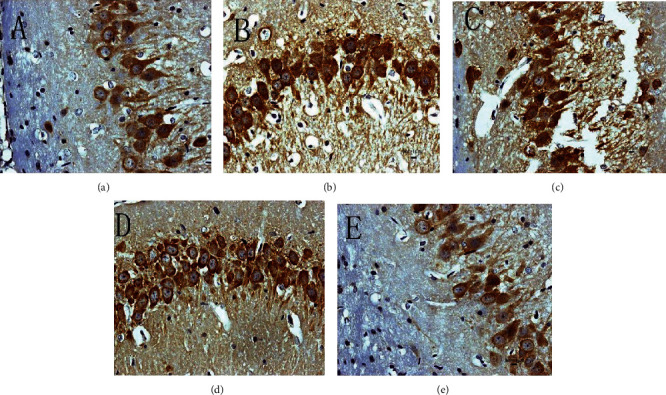
Expression of GRP78 protein in each group (72 hours after intervention. (a) The model group. (b) The HCC low-dose group. (c) The HCC medium-dose group. (d) The HCC high-dose group. (e) The nimodipine group).

**Figure 16:**
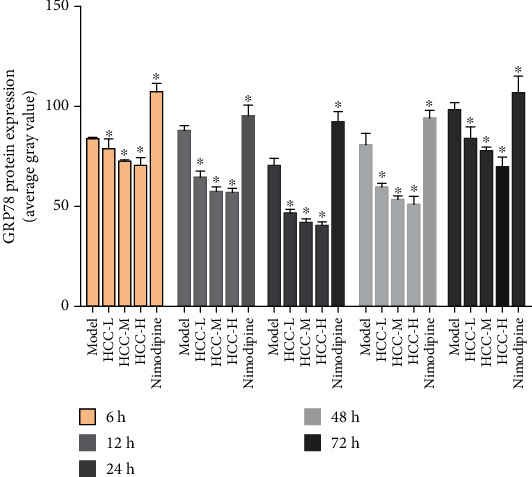
Average gray value of GRP78 after MCAO (^∗^compared with the model group, *P* < 0.05).

The average gray value of each group of animals was compared (gray value range 0-255; the larger the gray value is, the lower the expression level of the positive product is). GRP78-positive products are brown-yellow particles located in the cytoplasm of the neurons. Compared with the sham operation group, the GRP78 in the model group gradually increased the expression level from 6 hours after surgery, reached the peak at 24 hours, and was significantly downregulated after 72 hours (*P* < 0.05). After HCC intervention, the expression of GRP78 was significantly upregulated after operation, and the expression level was still higher than that of the model group and nimodipine group at 72 hours (*P* < 0.05) (Figures 14–16).

#### 3.5.2. Expression of p-PERK Protein

**Figure 17:**
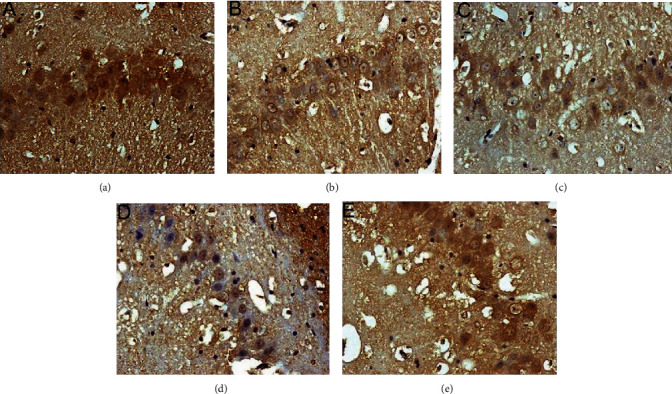
Expression of p-PERK protein in each group (24 hours after intervention. (a) The model group. (b) The HCC low-dose group. (c) The HCC medium-dose group. (d) The HCC high-dose group. (e) The nimodipine group).

**Figure 18:**
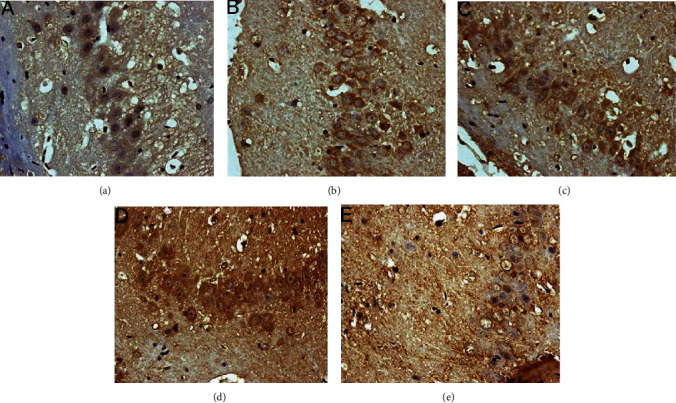
Expression of p-PERK protein in each group (72 hours after intervention. (a) The model group. (b) The HCC low-dose group. (c) The HCC medium-dose group. (d) The HCC high-dose group. (e) The nimodipine group).

**Figure 19:**
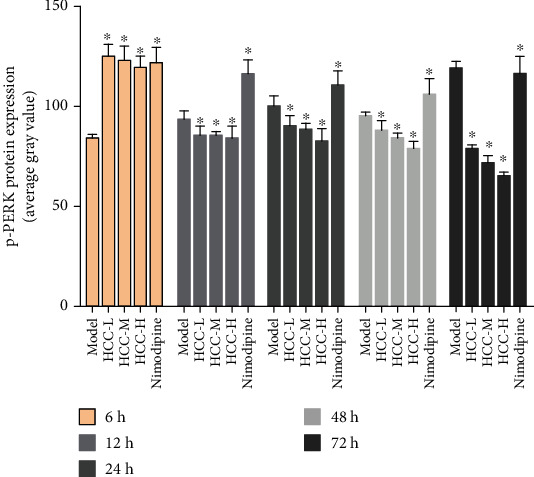
Average gray value of p-PERK after MCAO (^∗^compared with the model group, *P* < 0.05).

The p-PERK protein is brownish yellow granules, mainly expressed in the cytoplasm, and has almost no expression in the sham operation group. Compared with the sham operation group, the expression of MCAO in the model group increased at 6 hours after operation and then began to decrease; the difference was statistically significant (*P* < 0.05). After HCC intervention, the expression of p-PERK protein was significantly lower than that of the model group at 6 hours after surgery and then gradually increased; it was significantly higher than the model group and the nimodipine group after 12 hours and maintained at a high level (*P* < 0.05) (Figures 17–19).

#### 3.5.3. Expression of CHOP Protein

**Figure 20:**
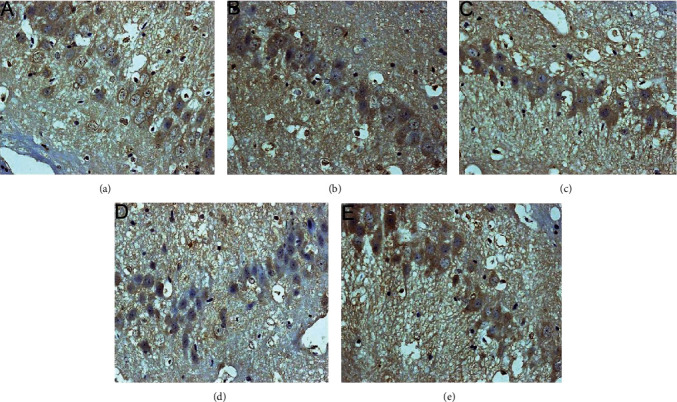
Expression of CHOP protein in each group (24 hours after intervention. (a) The model group. (b) The HCC low-dose group. (c) The HCC medium-dose group. (d) The HCC high-dose group. (e) The nimodipine group).

**Figure 21:**
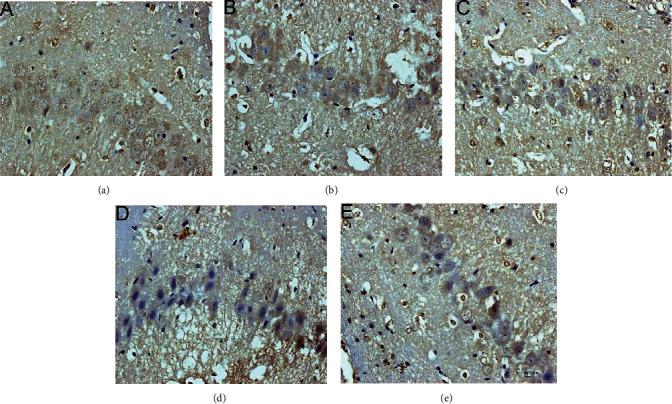
Expression of CHOP protein in each group (72 hours after intervention. (a) The model group. (b) The HCC low-dose group. (c) The HCC medium-dose group. (d) The HCC high-dose group. (e) The nimodipine group).

**Figure 22:**
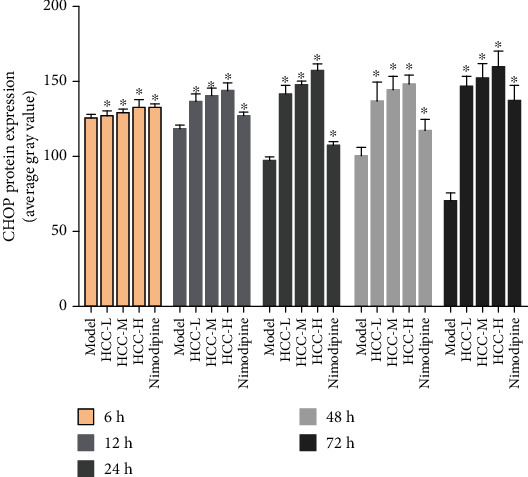
Average gray value of CHOP after MCAO (^∗^compared with the model group, *P* < 0.05).

The CHOP-positive product is a brownish granule located in the nucleus of the neuron. There was almost no expression in the sham operation group. Compared with the sham operation group, the expression level of the model group increased gradually from 6 hours after MCAO, and the difference was statistically significant (*P* < 0.05). Compared with the model group, the expression of CHOP after HCC intervention was significantly downregulated from 12 hours after MCAO, the difference was statistically significant (*P* < 0.05), and the expression of CHOP was significantly lower at 12 hours after the HCC intervention than in the nimodipine group (*P* < 0.05). (Figures 20–22).

### 3.6. The Expressions of GRP78, p-PERK, and CHOP Protein Detected by Western Blot

**Figure 23:**
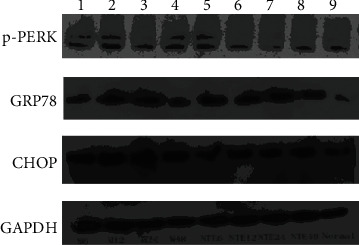
The expressions of GRP78, p-PERK, and CHOP protein detected by Western blot (1: 6 h after operation in the model group; 2: 12 h after operation in the model group; 3: 24 h after operation in the model group; 4: 48 h after operation in the model group; 5: 6 h after operation in the HCC group; 6: 12 h after operation in the HCC group; 7: 24 h after operation in the HCC group; 8: 48 h after operation in the HCC group; 9: the sham operation group).

**Figure 24:**
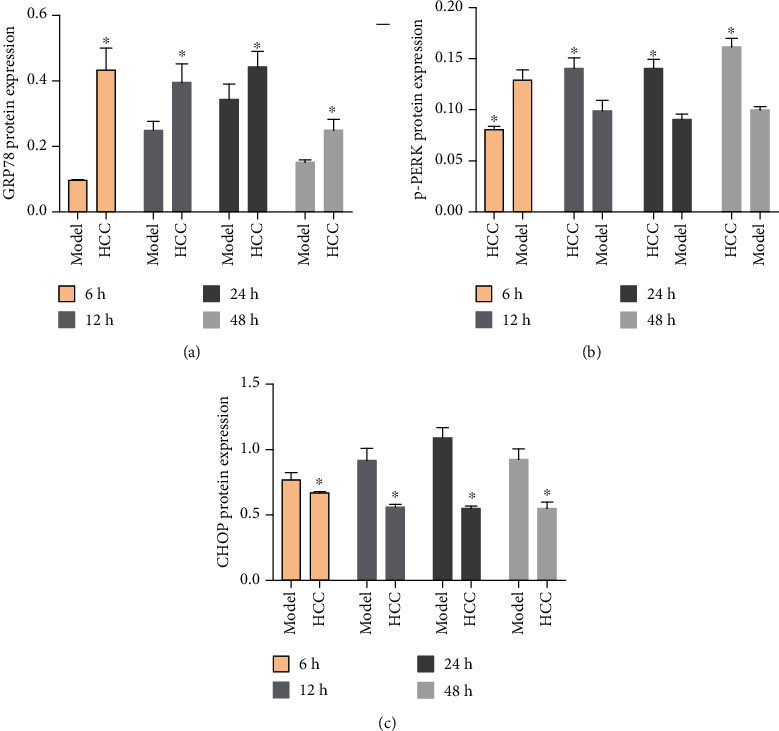
The relative expressions of GRP78, p-PERK, and CHOP protein detected by Western blot ((a) the expression of GRP78; (b) the expression of p-PERK; (c) the expression of CHOP. ^∗^Compared with the model group, *P* < 0.05).

Compared with the sham operation group, the expression of GRP78 protein in the model group was significantly upregulated, and the difference was statistically significant (*P* < 0.05). Compared with the model group, the expression of GRP78 protein was significantly upregulated at each time after MCAO intervention, and the difference was statistically significant (*P* < 0.05). Compared with the sham operation group, the expression of p-PERK protein in the model group was increased, and the difference was statistically significant (*P* < 0.05). Compared with the model group, the expression of p-PERK protein was lower than that of the model group at 6 hours after HCC intervention, but it was significantly upregulated from 12 hours, and the difference was statistically significant (*P* < 0.05). Compared with the sham operation group, the CHOP protein expression was upregulated in the model group, and the difference was statistically significant (*P* < 0.05). Compared with the model group, after HCC intervention, the expression of CHOP protein was significantly downregulated 12 hours after MCAO, and the difference was statistically significant (*P* < 0.05) (Figures 23 and 24).

### 3.7. The Expressions of GRP78 and CHOP mRNA Detected by RT-qPCR

**Figure 25:**
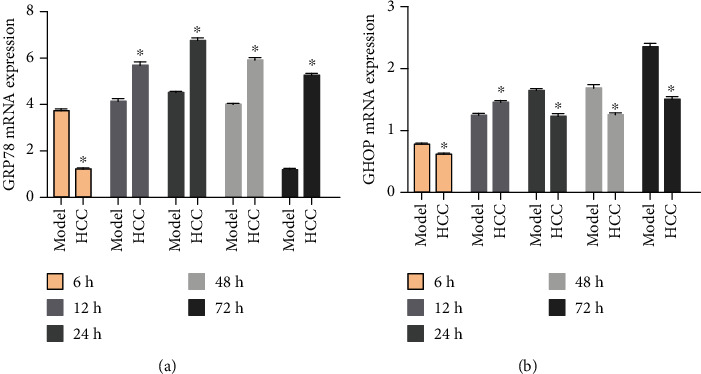
The expressions of GRP78 and CHOP mRNA detected by RT-qPCR ((a) the expression of GRP78; (b) the expression of CHOP. ^∗^Compared with the model group, *P* < 0.05).

The results showed that the specificity of GRP78 mRNA and CHOP mRNA amplification was higher. The relative expression levels of the GRP78 and CHOP genes calculated using CT values (relative to the sham group) showed the following: compared with the sham operation group, the expression of GRP78 mRNA in the model group was significantly upregulated, and the difference was statistically significant (*P* < 0.05); the expression of CHOP mRNA was also significantly upregulated after 12 hours, and the difference was statistically significant (*P* < 0.05). Compared with the model group, after HCC intervention, GRP78 mRNA expression was significantly upregulated 12 hours after MCAO; the difference was statistically significant (*P* < 0.05), while the expression of CHOP mRNA was downregulated 24 hours after MCAO (*P* < 0.05) (Figure 25).

The pathological process of ischemic cerebrovascular disease injury is extremely complicated, which involves energy metabolism disorder, excitatory amino acid toxicity, intracellular calcium overload, free radical damage, neuronal apoptosis, inflammatory response, and so on in brain tissue [53]. Oxidative stress and endoplasmic reticulum stress can occur simultaneously or sequentially during neuronal damage in IS [54], and calcium is the interconnected link between the two, eventually leading to nerve damage. The endoplasmic reticulum is the main place for cells to process proteins and store calcium. It is extremely sensitive to ischemia, hypoxia, and calcium balance disorders, which can result in endoplasmic reticulum stress and activate multiple signaling pathways [55]. After focal cerebral ischemia, the expression of the PRKK pathway protein in the hippocampal neurons changed significantly. Firstly, in the early stage of cerebral ischemia, GRP78 expression was evident in the neurons in hippocampal CA1 region, and the expression was highest after 24 hours of ischemia. Then, the expression was gradually reduced. After HCC intervention, GRP78 expression in the hippocampal CA1 neurons increased significantly [56]. Min et al. found that the expression of GRP78 in the IS rat neurons was significantly upregulated after 12 h after intervention with 2-deoxy-D-glucose [57]. Secondly, ischemia causes the activation of PERK (p-PERK) in the hippocampal neurons. This effect can be inhibited by HCC at 12 hours after surgery. PERK activation is an important pathway for ERS [58]. Gu et al. found that the mixed extract of Chuanxiong rhizoma and Radix Paeoniae Rubra can inhibit the expression of the PERSK protein of the ERS pathway and its downstream proteins eIF2*α* and CHOP, and then inhibit the apoptosis of the basal forebrain neurons in rats after MCAO [59]. In this study, the expression of CHOP in the neurons caused by PRK activation can also be downregulated by HCC. However, the time of inhibition of CHOP protein and genes is not the same, which requires further experiments to explore its mechanism. Cao et al. found that Yiqi Fumai (YQFM) powder, an extract of Shengmai powder, can inhibit the expression of the ERS pathway marker protein CHOP [60]. Our research also shows that HCC has a certain effect on improving the behavior of rats after IS, and it can significantly increase the survival of the hippocampal CA1 neurons after MCAO. HCC can inhibit the expression of p-PERK in the PERK pathway, significantly promote the expression of GRP78 protein, and inhibit the expression of CHOP protein postoperatively, especially at 24 h postoperatively. The results of RT-qPCR also showed that HCC can significantly reduce the expression of CHOP in the hippocampal CA1 neurons 72 h after MCAO.

## 4. Conclusions

HCC may achieve a role in the treatment of IS by intervening in a series of targets (such as ALB, AKT1, MMP9, IGF1, and CASP3), biological processes (such as endoplasmic reticulum stress, inflammation modules, hypoxia modules, regulation of neuronal apoptosis and proliferation, angiogenesis, coagulation and platelet activation, and oxidative stress), and signaling pathways (such as PI3K-Akt, FoxO, TNF, HIF-1, and Rap1 signaling). The animal experiments also verified that HCC can improve the neurobehavioral scores and protect the neurons of IS rats, and regulate the expressions of endoplasmic reticulum stress-related targets [GRP78 (HSPA5), p-PERK (EIF2AK3), and CHOP (DDIT3)].

## Figures and Tables

**Table 1:** The herb of HCC extract.

Herb	Ratio	Place of origin	Specimen number
*Hedysarum multijugum Maxim.*	4	Gansu	2014062101
*Chuanxiong rhizoma*	1	Sichuan	2014062410
*Pheretima*	1.5	Guangxi	2014062205
*Bombyx batryticatus*	1.5	Zhejiang	20140622307

**Table 2:** Mobile-phase elution gradient.

Time	A
0	95
15	85
35	70
40	60
50	60
65	80
70	95
80	95

**Table 3:** Amplification primer sequence of CHOP, GRP78, and *β*-actin.

Primer	Sequence	Annealing temperature	Length
CHOP	Forward: 5-CACTCTTGACCCTGCTTCTC-3 Reverse: 5-TCTTCCTCCTCTTCCTCCTG-3	60	116 bp
GRP78	Forward: 5-GCACAGACGGGTCATTCCAC-3 Reverse: 5-CCTATGTCGCCTTCACTCC-3	59	120 bp
*β*-Actin	Forward: 5-CTCAAGAAGGAGCGGTTGGT-3 Reverse: 5-CCAAGTGCAGGAACGAGTCT-3	60	166 bp

**Table 4:** Cluster of HCC-IS PPI network.

Cluster	Score	Nodes	Edges	Targets and genes
1	54.747	76	2053	TLR2, INS, VCAM1, SERPINE1, CCR5, APOE, BCL2L1, TGFB1, VWF, NGF, TLR8, TLR7, PLG, TLR4, EDN1, CD40LG, CASP1, CASP3, CX3CL1, PPARG, CAT, HMOX1, HMGB1, CCL5, HRAS, IL18, HSPA4, PECAM1, CREB1, CYCS, IL6, JAK2, REN, TNF, ADIPOQ, IL1R1, SELE, SELP, CTLA4, IL10, MAPK1, IL1B, CSF3, MAPK14, MAPK8, ACE, SRC, ICAM1, AGT, CCR2, IL4, EGFR, ELANE, MMP1, BDNF, NLRP3, MMP2, ELN, MMP3, MMP9, SELL, AKT1, PTGS2, CD40, ALB, NOS2, NOS3, IL6R, ANXA5, VEGFA, CXCL8, TP53, MPO, SPP1, CCL2, CSF2
2	20.475	81	819	IL1A, CRP, CCR3, PIK3CA, CCL11, PGF, RETN, GRB2, PGR, GSK3B, AGTR1, PLAU, TNFRSF11B, F3, ACTN4, PDGFRB, CCNA2, HPGDS, CD209, ENG, CDC42, LOX, HSP90AA1, PTPN11, PF4, CFD, IGF1, MCL1, IGF1R, IL2, SERPINF2, PPBP, RAF1, ITGAL, KNG1, NFE2L2, ITGB1, KDR, RHOA, CTSB, AIF1, KIT, LCK, LCN2, AGER, LGALS3, MAP2K1, MAPK10, NOTCH1, SOD2, SPARC, MDM2, F13A1, IL4R, MET, ADAM17, STAT1, IL13, MIF, IL1RN, MMP13, SYK, SMAD3, IL9, ESR1, TEK, MMP7, TGFB2, F8, AKT2, ALDOA, MMRN1, FCGR2A, EGF, FGF1, NR3C1, AR, CXCL12, ARG1, XIAP, CDKN2A
3	9.714	64	306	BACE1, GM2A, FGA, BPI, APOH, BRAF, LPA, BTK, OLR1, GSTP1, COG2, CANT1, HEXB, CASP7, CYBA, CDA, F2R, HSPA1A, HSPA8, FGB, QPCT, CHIT1, RAC1, HP, IMPDH1, IGFBP3, INSR, ASAH1, RNASE2, RNASE3, CTSG, CTSL, ITGB3, CTSS, LTA4H, LTF, SELPLG, MAN2B1, SERPINA1, ITGA2, C3, MAPT, AHSG, EIF4E, F12, MMP12, LPL, AKR1B1, F10, F11, TGM2, F2, F7, CHI3L1, FABP4, FABP5, ANG, NAMPT, FGG, ZAP70, F5, ARSA, ATIC, APOA1
4	8.338	66	271	FBN1, ITIH4, LDLR, S100B, ADRA2A, BMP2, NOS1, PIK3R1, GSR, GFAP, APOB, SERPINC1, PLAT, NPY, THBD, P2RY12, PRL, CST3, MBP, PSAP, CDK2, PTK2, PTPN1, ATF6, CDK6, XBP1, MAP3K5, C5, CHEK1, DDIT3, BCL2L11, SERPIND1, PCSK9, AGTR2, ATF4, EIF2AK3, HSPA5, RBP4, ABCB1, GDNF, JAK3, TF, PROC, ALOX5, ABL1, TWIST1, ITGB2, CP, SOD1, APOA5, ERBB4, ESR2, APLNR, MMP8, TREM1, JAG1, NQO1, SAA1, CFLAR, TTR, APAF1, APOA2, VDR, PARP1, GC, PTX3
5	4.294	35	73	UCP2, CTSK, CD14, ADRB2, HABP2, F13B, PDPK1, PON1, ABCA1, CETP, PIK3CG, CALCA, ADM, PLA2G7, LIPC, HCK, PROZ, SORT1, PPARA, HK1, HMGCR, BCL2, PROCR, MBL2, RAC2, APCS, G6PD, ITGA2B, CRYZ, CPB2, TFPI, CSK, ENTPD1, APOC3, PAPSS1
6	4	5	8	GNPDA2, GALE, GALK1, UAP1, GNPDA1
7	3.667	7	11	DHODH, MT-CO2, MT-CYB, UQCRFS1, GART, SHMT1, DHFR
8	3.636	23	40	ACVRL1, GLO1, PDE4B, RXRA, PDE4D, MTHFD1, PSPH, BHMT, NR1H2, TYMP, ACADM, UCK2, UMPS, HADH, NT5M, FOLH1, IVD, PNP, ARG2, OTC, ADK, GATM, AHCY
9	3.556	10	16	NAGS, LDHB, SORD, CLPP, ME2, SRM, ALAD, OAT, TPI1, FECH
10	3.429	15	24	PRKACA, GSS, TNNI3, GSTA1, GSTA3, GSTM1, NR1H4, NPPB, RARA, SMARCA4, GPX3, KAT2B, AGXT, NPPA, MTHFR
11	3.333	10	15	CYP4A11, CYP3A5, GSTM2, CYP4F2, CYP2J2, GSTO1, HSD17B1, STS, ADH1B, ADH1C
12	3.333	7	10	CYP1A1, NR1I3, NR3C2, SULT1A1, SULT1E1, CYP17A1, CYP11B2
13	3.333	7	10	CYP2C19, NR1I2, RXRB, THRB, RARB, CYP2C8, RARG
14	3.25	9	13	GCK, IMPDH2, APRT, DTYMK, GMPR, GMPR2, TK1, PYGL, PCK1
15	3	5	6	HADHA, ACADSB, PCCA, PCCB, GCDH
16	3	3	3	AMY1B, AMY1C, AMY1A
17	2.889	10	13	AURKA, PRKG1, DUT, PLK1, HTR2A, HSP90AB1, ACTA2, P2RY1, TBXA2R, MAPK12
18	2.5	9	10	BID, KALRN, EDNRA, BBC3, ERN1, TXNIP, RAB5A, AVP, BAK1

## Data Availability

The data used to support the findings of this study are included within the article and the supplementary information files.

## Supplementary Materials

Supplementary Materials Table S1: compound targets for each compound of HCC. Table S2: IS genes. Table S3: enrichment analysis of clusters based on Gene Ontology (GO) annotation of HCC-IS PPI network. Table S4: pathway enrichment analysis of HCC-IS PPI network.
